# Molecular characterization of thioredoxin peroxidases 1 from *Trichinella spiralis* and its potential in generating protective immunity

**DOI:** 10.1186/s13071-026-07517-8

**Published:** 2026-06-20

**Authors:** Mingwei Tong, Zixin Guo, Mianjing Wang, Yong Yang, Mengxiao Fu, Yuchen Gao, Jie Ren, Zhixin Wang, Peixia Yu, Xin Zhou, Hongli Liu, Hailong Wang, Hairu Wang

**Affiliations:** 1 School of Basic Medical Sciences, Shanxi Medical University, Jinzhong, 030600 China; 2 Department of Laboratory Medicine, Shanxi Bethune Hospital, Shanxi Academy of Medical Sciences, Third Hospital of Shanxi Medical University, Tongji Shanxi Hospital, Taiyuan, 030032 China; 3 College of Veterinary Medicine, Shanxi Agricultural University, Taigu, Jinzhong, 030801 China; 4 Shanxi Key Laboratory of Metabolic-Immune Homeostasis and Drug Innovation, Shanxi Medical University, Jinzhong, 030600 China

**Keywords:** *Trichinella spiralis*, Thioredoxin peroxidases, Immune protection, Vaccine candidate, Immunization

## Abstract

**Background:**

*Trichinella spiralis* remains a significant food-borne parasitic threat with no licensed vaccine available. This study evaluated the vaccine potential of *T. spiralis* thioredoxin peroxidase 1 (Ts-TPx1).

**Methods:**

Recombinant Ts-TPx1 (rTs-TPx1) was expressed, purified, and characterized. Mice were immunized intraperitoneally with rTs-TPx1 plus ISA206, ISA206 alone, or PBS, and subsequently challenged orally with *T. spiralis* larvae. Systemic and intestinal immune reponses, protection efficacy, intestianl barrier function, and pathological changes in the murine intestine and diaphragm muscle were subsequently assessed.

**Results:**

Ts-TPx1 belongs to the 2-Cys peroxiredoxin  family. Native Ts-TPx1 is expressed across key lifecycle stages, predominantly localizing to the parasite surface and stichosome. rTs-TPx1 immunization elevated serum levels of Ts-TPx1-specific IgG, IgG1, and IgG2a, with IgG1 levels higher than IgG2a, and increased splenocyte secretion of specific INF-γ and IL-4. The vaccine conferred significant protection, reducing intestinal adult worm burden by 65.0% and muscle larval burden by 61.8%. It also enhanced intestinal mucosal immunity, as evidenced by increased sIgA and Muc2 levels, and ameliorated intestinal and diaphragm muscle pathology.

**Conclusions:**

rTs-TPx1 immunization elicited specific intestinal mucosal immunity and Th2-biased mixed Th1/Th2 systemic protective response, establishing it as a promising vaccine candidate against *Trichinella* infection.

**Graphical Abstract:**

**Figure:**
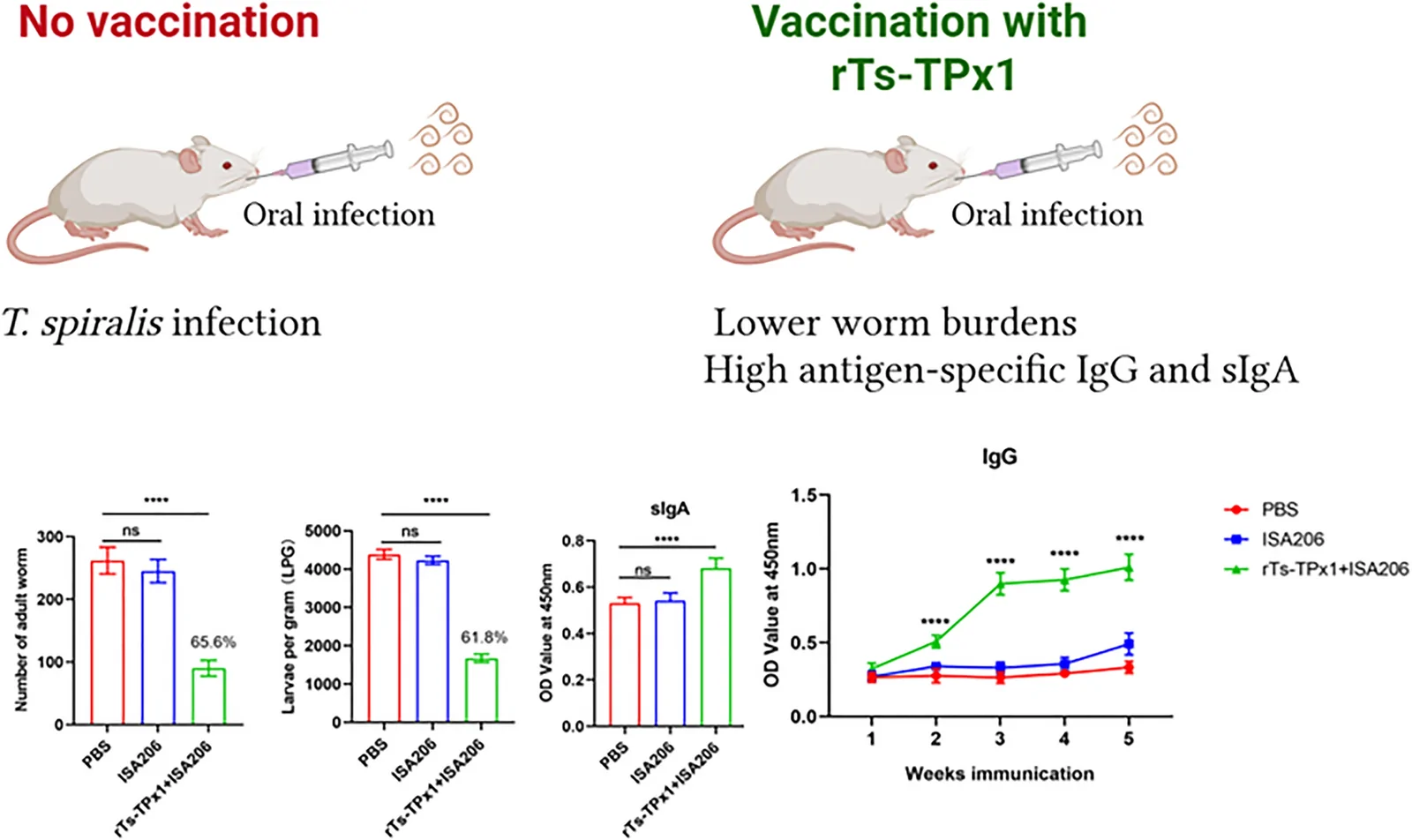

## Background

*Trichinella spiralis* (*T. spiralis*) is an important food-borne parasite that infects more than 150 species of mammalian hosts throughout the world, and is responsible for the zoonotic disease trichinellosis. Accidental ingestion of raw or semi-cooked meat containing muscle larvae (ML) of *T. spiralis* results in human infection [1]. Historically, meat from domestic pigs has been considered the main source of human *T. spiralis* infection in China and other developing countries [2]. Human trichinellosis due to ingesting meat of other animals infected by nematode larvae of the genus *Trichinella*, such as game animals (e.g., bears, cougars, foxes, walruses, and wild pigs), dog, and soft-shelled turtle meat, has been reported [3–5], which has greatly increased the risk of human infection. In European Union, 41 confirmed cases of human trichinellosis were recorded in the 22 member states only in 2022 [6]. From 2009 to 2020, 8 outbreaks of human trichinellosis, with 479 cases and 2 deaths, were reported in China [7]. *T. spiralis* infection causes a severe public health problem and has become a great threat to meat food safety. Therefore, there is an urgent need for developing effective vaccines to prevent *T. spiralis* infection in food animals.

After ingestion of the encapsulated ML-infected meat by a new host, the larvae are released from their collagen capsules and activated to intestinal infective larvae. These larvae penetrate intestinal epithelial cells and develop into adult worms (ADs). In intestine, female adults produce newborn larvae, which migrate into the circulatory system, invade the muscles and grow into ML, completing the lifecycle [8, 9]. During *T. spiralis*’s lifecycle, several molecules of the parasites involved in driving invasion, migration, parasite survival, or immunity have been identified and developed as vaccine candidates [10–12]. Although some of these vaccine candidates have shown positive effects against infection, no commercial vaccine is currently available. Characterization of these molecules and exploration of their functions should be intensified to better understand parasite–host interaction and develop vaccines against *Trichinella* infection.

Thioredoxin peroxidases (TPx), a member of the peroxiredoxin superfamily, acts as antioxidant enzymes by reducing hydrogen peroxide [13]. TPx exists in almost all organisms, and appears to be abundant in helminth parasites. TPx could deoxidize H_2_O_2_ into water in a system comprised by thioredoxin, thioredoxin reductase and NADPH, thereby protecting helminths against oxidative damage. During invasion and rapid growth phases of *T. spiralis*, the host immune cells, such as macrophages and neutrophils, generates large amounts of reactive oxygen species (ROS) to attack the parasite. By neutralizing these cytotoxic ROS, TPx directly helps *T. spiralis* defend against ROS-mediated oxidative assault, thereby facilitating initial infection establishment and long-term survival. This essential role in counteracting non-specific oxidative damages distinguishes TPx from many other secreted proteins such as proteases that may function primarily in invasion or immune evasion, and makes it a rational target for vaccine development [14]. Candidate vaccines based on TPx homologs have been evaluated in *T. spiralis*, filarial nematodes, and trematodes. Although TPxs have been extensively characterized in several helminth parasites [15–17], limited information is available for *T. spiralis*. Zhang et al. first cloned three members of Tpx from *T. spiralis* (TsTPX1–3) [18], and adoptive transfer of TsTPX2-regulated macrophages exhibited a 44.7% reduction in adult worm burden following challenge with *T. spiralis* infective larva [19]. However, the protective role of Tpx from *T. spiralis* remains underexplored.

Herein, we characterized the TPx1 gene product of *T. spiralis* (Ts-TPx1). The expression patterns in *Trichinella* developmental stages and the immune protection against *T. spiralis* infection in mice triggered by recombinant Ts-TPx1 (rTs-TPx1) were systematically assessed. The results will assist in interpreting the parasite–host interaction and providing evidence for TPx as a promising vaccine candidate against *Trichinella* infection.

## Methods

### Parasites and animals

The *T. spiralis* (ISS534) parasites were maintained by serial passage in mice in our department. ML of *T. spiralis* were collected from infected mice at 35 day post infection (dpi) and ADs were collected from the intestine at 6 dpi (AD6) as previously reported [20, 21]. Four-week-old female ICR mice were purchased from the SPF (Beijing) Biotechnology Co., Ltd (Beijing, China) and raised in individually ventilated cages under specific pathogen-free conditions. Animals were treated in strict accordance with the National Institutes of Health guidelines (publication no. 85-23, revised 1996). Animals were reviewed and approved by the Ethical Committee of Shan Xi Agricultural University affiliated with the Provincial Animal Health Committee, Shan Xi Province, China (Ethical Clearance number No. SXAU-EAW-2025M.LP.011014357).

### Preparation of rTs-TPx1

The complete coding sequence (CDS) of Ts-TPx1 gene was cloned according to the CDS of *T. spiralis* (GenBank Accession No. XM_003375787), and then the acquired sequence was subcloned into the pCold I vector (TaKaRa, China). The recombinant expression plasmid was transfected into *Escherichia coli* strain BL21 (DE3). Expression of rTs-TPx1 protein was induced using 0.2 mM IPTG for 16 h at 20 °C. rTs-TPx1 was purified using Ni-affinity chromatography (APExBIO, China). Purified rTs-TPx1 was analyzed on 12% sodium dodecyl sulfate–polyacrylamide gel electrophoresis (SDS–PAGE).

### Production of anti-rTs-TPx1 serum

For antisera production, five mice were immunized intraperitoneally with 30 μg rTs-TPx1 protein in 50 μl sterile PBS, emulsified with an equal volume of ISA206 adjuvant. Two booster immunizations were carried out using the same protocol at a 2-week interval. Blood samples were collected 2 weeks after the last immunization, and anti-rTs-TPx1 serum was isolated and stored at −80 °C until use. Serum was also collected from mice experimentally infected with 300 *T. spiralis* ML at 35 dpi (infection serum) and normal pre-immune serum was obtained before immunization.

### Western blot analyses of Ts-TPx1 expression

Crude antigens from AD6 and ML, and rTs-TPx1 were separated by 12% SDS–PAGE and transferred to a polyvinylidene difluoride (PVDF) membrane (Millipore, USA), respectively. After blocking in 5% skim milk at 37℃ for 1.5 h, the membrane was incubated at 37℃ for 1 h with 1:500 diluted anti-rTs-TPx1 serum, infection serum, and pre-immune serum. After washing five times with 10 mM tris-buffered saline (PH 7.6) containing 0.05% Tween (TBST), the strips were incubated with HRP-conjugated goat anti-mouse IgG antibody(1:80,000, Sigma, USA) and finally detected by the enhanced chemiluminescence (ECL) method with Meilunbio^®^ fg super sensitive ECL luminescence reagent (Meilunbio, China).

### Immunolocalization of Ts-TPx1 at various developmental stages of *T. spiralis*

Fresh intact ML and AD6 worms were fixed with in neutral formalin for 24 h, embedded in paraffin, and cut into 4-μm-thick cross sections. Then, the immunofluorescence assay (IFA) was carried out to observe the expression and localization of native Ts-TPx1 in *T. spiralis* at different stages as previously reported [22]. In brief, following deparaffinization and permeabilization, worm cross sections were blocked with 5% normal goat serum at 37 °C for 30 min and incubated overnight at 4 °C with a 1:50 dilution of three different sera (anti-rTs-TPx1 serum, infection serum, and pre-immune serum). After washing three times, sections were stained with Cy3-labeled Goat Anti-Mouse IgG (H + L) (1:100, Beyotime, China) for 1 h at room temperature and were subsequently incubated with 1 μg/ml DAPI (Beyotime, China) for 5 min. The sections were observed under fluorescent microscope (AxioObser Z1, Germany).

### Immunization with rTs-TPx1 and challenge experiments

To determine the immune protection induced by rTs-TPx1 immunization, 69 female ICR mice were randomly divided into three groups (23 mice per group): (1) PBS group, (2) ISA206 group, and (3) rTs-TPx1 + ISA206 group. Mice in rTs-TPx1 + ISA206 group were vaccinated intraperitoneally with 30 μg of rTs-TPx1 emulsified in ISA206. Two booster immunizations were performed using the same method at an interval of 2 weeks. The other two groups of mice were administrated with an equal volume of PBS alone or PBS emulsified with ISA206 at the same time as the rTs-TPx1 + ISA206 group received the rTs-TPx1 vaccination. One week after the final boost vaccination, all mice were challenged orally with 300 *T. spiralis* ML. 6 mice of each group were euthanized at 6 and 35 dpi, respectively. The intestine at 6 dpi and the diaphragm muscles at 35 dpi were obtained, and the number of intestinal AD6 or muscle larvae per gram (LPG) was determined. The protection rate was calculated as follows: adult worm reduction (%) = [(Mean worm burden in control group − Mean worm burden in vaccinated group)/Mean worm burden in control group] × 100. The reduction in muscle larval burden was calculated using the formula: reduction (%) = [(Mean LPG of control group − Mean LPG of vaccinated group)/Mean LPG of control group] × 100. All counts were performed blinded. Another 6 mice in each group were euthanized at 6 dpi, and a 20-cm-long intestinal segment was collected from each mouse. The additional mice in each group were sacrificed at 1 week (prior infection) after the last immunization for cytokines detection.

### Histological examination of intestines and muscles from mice

The intestines were collected from mice at 6 dpi and the diaphragm muscles were collected from mice at 35 dpi. The tissue samples were fixed overnight in neutral formalin, paraffin-embedded, sectioned at 4-μm thickness, and processed for staining. Hematoxylin and eosin (HE) staining was performed according to standard protocols [23] to observe the morphological changes and the severity of inflammatory cells infiltration in intestine and diaphragm muscle tissues by light microscopy (Olympus, Tokyo, Japan). The inflammatory cells from three groups of mice were analyzed by a semi-automated area-based measurement using Image J (1.53).

### Analysis of the intestinal goblet cells and mucus layers

The 4-μm thickness sections of intestine tissues were prepared. Alcian blue staining was carried out to quantify the goblet cell number according to the kit manufacturer instructions (Solarbio, China). For immunofluorescence assays, sections were incubated with mouse anti-mucin 2 (Muc2) antibody (1:100, cohesion biosciences, China). The sections were then washed with PBS and treated with a Cy3-conjugated goat anti-mice secondary antibody (1:200, Beyotime, China) for 1 h at room temperature, and were observed under a microscope (AxioObser Z1, Germany).

### Analysis of specific antibody responses by indirect ELISA

Specific antibodies against rTs-TPx1 were assayed. At weeks 0, 2, 4, 6 and 8 after the first vaccination, 200 μl of tail blood was collected from vaccinated mice, and serum samples were isolated and preserved at −80 °C until use. The titers of anti-rTs-TPx1 IgG, IgG1, and IgG2 antibodies were measured by indirect enzyme-linked immunosorbent assay (ELISA). In brief, plates were coated with 2 μg/ml rTs-TPx1 at 4 °C overnight. After blocking with 5% skimmed milk at 37 °C for 2 h, the plates were incubated with serial dilutions of 1:200 dilutions of immune sera, followed by incubation with horseradish peroxidase (HRP)-conjugated goat anti-mouse IgG (1:80,000; Sigma-Aldrich, USA). For IgG subtype analysis, plates were incubated with goat anti-mouse IgG1 (1:5000; cohesion biosciences, China), or IgG2a (1:5000; cohesion biosciences, China) at 37 °C for 1 h, followed by incubating with HRP-conjugated rabbit anti-goat IgG (1:5000 dilution, Elabscience, China).Finally, the plates were washed, and the enzyme reaction was developed with 100 μl of 3,3′,5,5′-tetramethylbenzidine substrate (TMB; Nanjing jiancheng bioengineering institute). After incubation in dark at room temperature, the reaction was stopped by addition of 50 μl of 12% sulphuric acid, and the optical density (OD) at 450 nm was detected using a microplate reader (Tecan, Schweiz, Switzerland).

To assess rTs-TPx1-specific mucosal sIgA in the gut fluids, 20-cm-long intestinal segments were obtained. Cold PBS with 1% protease inhibitor was used to wash the intestine interior, and the washing fluid was gathered for centrifugation at 12,000×*g* for 5 min at 4 °C to obtain the purity of intestine fluids. rTs-TPx1-specific sIgA was determined by ELISA with 2 μg/ml of rTs-TPx1 as the coating antigen. After incubating by HRP-conjugated goat anti-mouse IgA secondary antibody (cohesion biosciences, China), TMB substrate was incubated in the dark at 37 °C for 10 min and the OD_450_ values were determined as described elsewhere [24].

### Cytokine assays

In order to assess rTs-TPx1-specific cellular immune responses, the cytokine profile from splenocyte culture supernatants was determined as described previously [25]. In brief, 1 week after the last immunization, five mice of each group were sacrificed, and splenocyte suspensions were prepared. 2 × 10^6^ cells were cultured in RPMI 1640 with 10% fetal bovine serum (FBS), penicillin (100 U/ml), and streptomycin (100 μg/ml) in a 24-well cell culture plate. The cells were stimulated with 5 μg/ml rTs-TPx1 for 48 h at 37 °C, supernatants were collected and the levels of cytokines IFN-γ and IL-4 were measured with ELISA assay according to the manufacturer’s instructions (Cusabio, China).

### Statistical analysis

The data were analyzed using the GraphPad Prism 8.0 (GraphPad InStatt Software, San Diego, California). The data were expressed as the mean ± standard deviation (SD), and differences between the groups were compared by one-way ANOVA or two-way ANOVA. *p* values were expressed as * *P* < 0.05, ** *P* < 0.01, *** *P* < 0.001, and **** *P* < 0.0001.

## Results

### Bioinformatic analysis of Ts-TPx1 gene

**Fig. 1:**
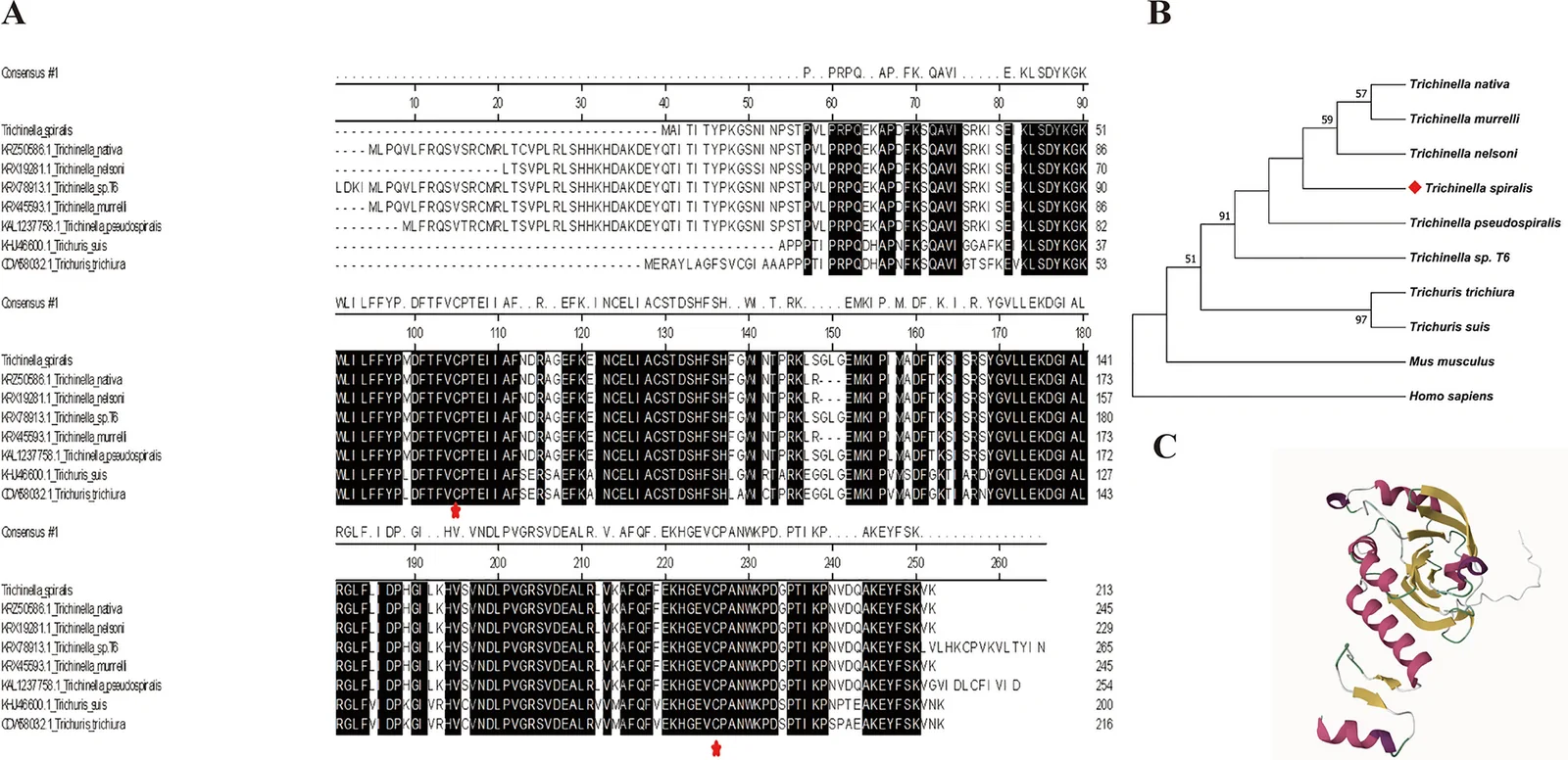
Sequence alignment, phylogenetic tree, and the predicted three-dimensional structure. **A** Multiple sequence alignment of Ts-TPx1 and homologs from other *Trichinella* species and other organisms. Asterisks represent conserved cysteine residues. **B** Phylogenetic tree consisting of TPx from 10 organisms determined with the NJ method. **C** Predicted three-dimensional structure of the Ts-TPx1 protein. α-Helices are shown in pink, β-sheets in yellow, and random coils in green

The complete Ts-TPx1 cDNA sequence was 642 bp and encodes 213 amino acids (aa) with a predicted molecular weight of 24 kDa and isoelectric point of 8.62. Homology comparison using the deduced amino acid sequence indicated that Ts-TPx1 shared identities of 97.6–98.6% with thioredoxin peroxidase from other 5 *Trichinella* species (*T. nelsoni*, *T. nativa*, *T.sp. T6*, *T. murrelli*, and *T. pseudospiralis*) and identities of 73.2–77.9% with the thioredoxin peroxidase from other 2 nematode species (*Trichuris suis* and *Trichuris trichiura*). Furthermore, amino acid sequence alignment of Ts-TPx1 shared two highly positionally conserved cysteine residues (Cys-66 and Cys-187, as indicated by asterisk in Fig. 1A, respectively) with orthologs from other nematodes, suggesting Ts-TPx1 to be a member of the 2-Cys peroxiredoxin family (Fig. 1A).

Phylogenetic analysis of Ts-TPx1 homologs was shown in Fig. 1B, which strongly supported the presence of a monophyletic group consisting of the genus *Trichinella*. Within the genus *Trichinella*, Ts-TPx1 was genetically relevant to the thioredoxin peroxidase from *T. nativa*, *T. murrelli*, and *T. nelsoni*, and had a closer evolutionary relationship with nematode TPx than vertebrate TPx. 3D structure prediction was performed using the Swiss model, confirming the presence of α-helices, β-sheets, and random coils  (Fig. 1C).

### SDS‑PAGE and western blot identification of the rTs-TPx1 protein

**Fig. 2:**
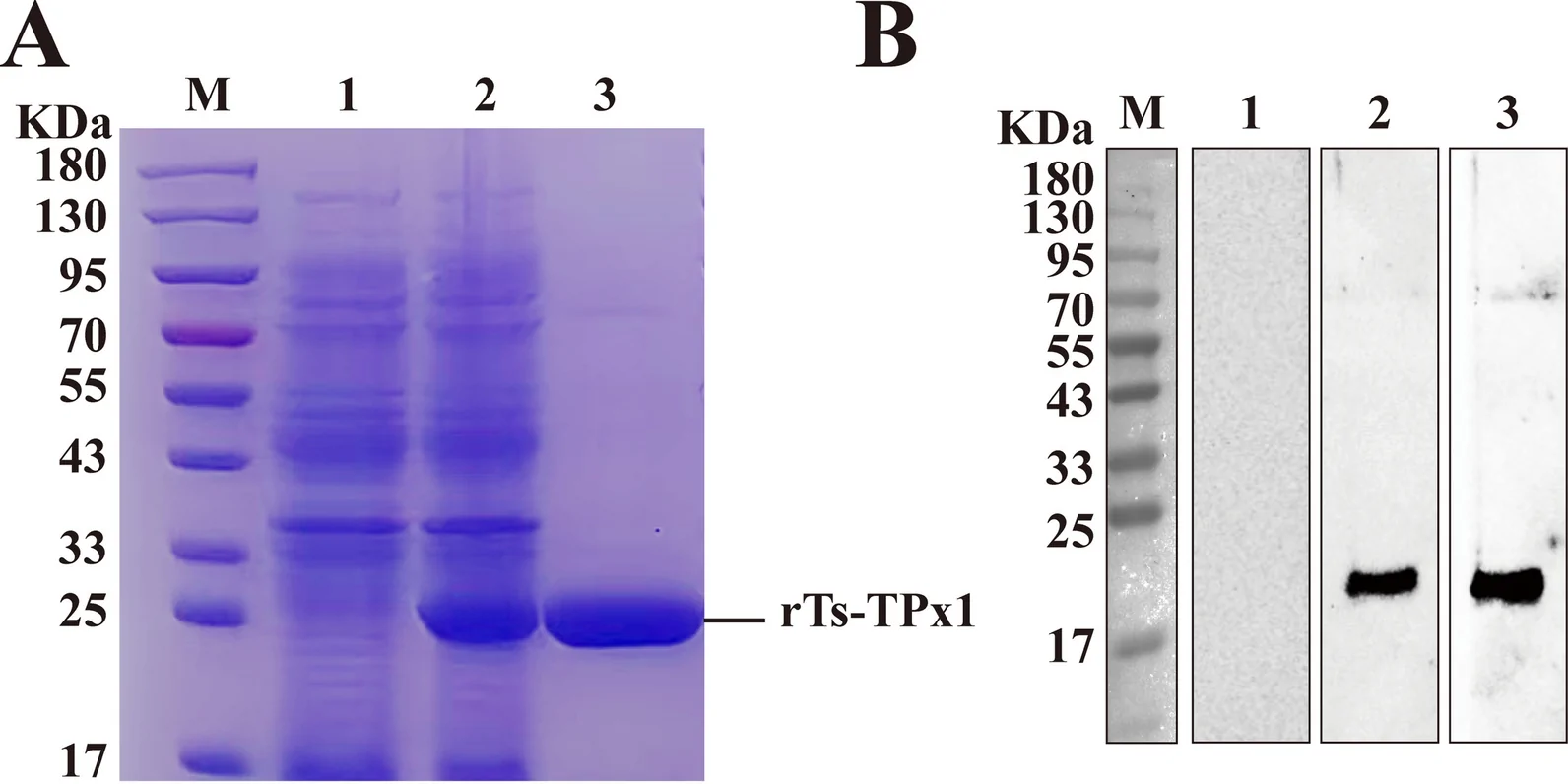
Purification and identification of rTs-TPx1. **A** SDS–PAGE analysis of the purified rTs-TPx1. Lane M: protein marker, lane 1: lysate of BL21 (DE3) bacteria transfected with pCold I-TPx1 prior to induction, lane 2: lysate of BL21 (DE3) bacteria transfected with pCold I-TPx1 following induction, lane 3: purified rTs-TPx1. **B** Western blot analysis of rTs-TPx1 antigenicity. Lane 1: rTs-TPx1 incubated with pre-immune murine serum, lane 2: rTs-TPx1 incubated with *T. spiralis*-infected murine serum, lane 3: rTs-TPx1 incubated with anti-rTs-TPx1 murine serum

Soluble rTs-TPx1 was successfully obtained and identified as an approximately 24 kDa single band by SDS–PAGE analysis after purification (Fig. 2A). Western blot assays indicated that the rTs-TPx1 protein was specifically recognized by the infection serum and anti-rTs-TPx1 murine serum but not by the normal pre-immune serum (Fig. 2B). These results demonstrated that the Ts-TPx1 of *T. spiralis* is an immunodominant antigen, and the recombinant protein expressed in *E. coli* was antigenic.

### Native Ts-TPx1 is expressed in different life stages of *T. spiralis*

**Fig. 3:**
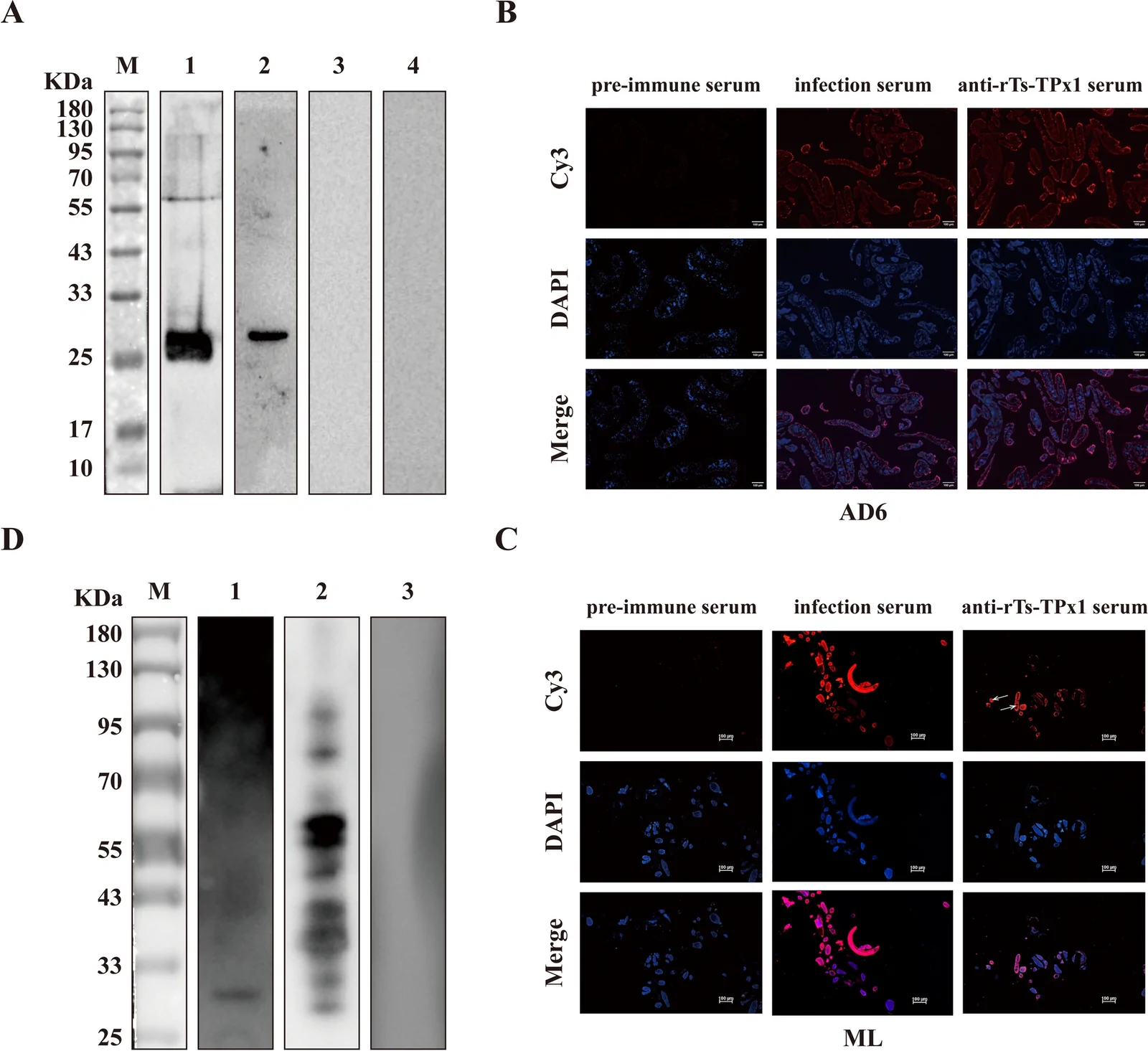
Analysis of the expression pattern and immunolocalization of Ts-TPx1 at various developmental stages of *T. spiralis*. **A** Western blot analysis of proteins from ML and AD6 by anti-rTs-TPx1 serum or pre-immune serum. Lane M: protein marker, lane 1: somatic extract antigens of ML incubated with anti-rTs-TPx1 murine serum, lane 2: somatic extract antigens of AD6 incubated with anti-rTs-TPx1 murine serum, lane 3: somatic extract antigens of ML incubated with incubated with pre-immune murine serum, lane 4: somatic extract antigens of AD6 incubated with pre-immune murine serum. **B**, **C** Sections of AD6 and ML were probed by different murine sera. AD6 and ML probed using pre-immune serum and *T. spiralis*-infected serum (infection serum) were used as negative and positive controls, respectively. Arrows indicated the positive immunofluorescence signal at the stichosome of ML. Scale bars: 100 μm. **D** Western blot analysis of proteins from ML ES by anti-rTs-TPx1 serum. Lane 1: ES ML incubated with anti-rTs-TPx1 murine serum, lane 2: ES ML incubated with serum from mice infected with 300 ML of *T. spiralis* and collected at 40 dpi, lane 3: ML ES incubated with pre-immune murine serum

To determine the expression and distribution of native Ts-TPx1 in *T. spiralis* at various developmental stages, including ML and AD6, western bolt and IFA analyses were performed using different murine sera. Western bolt results showed that the anti-rTs-TPx1 polyclonal antibodies could specifically recognize native Ts-TPx1 in somatic extract antigens from AD6 and ML (Fig. 3A). IFA results revealed that Ts-TPx1 was present on the epicuticle of AD6 and ML (Fig. 3B, C). Strong immunofluorescence signals were also visible at the stichosome of ML at 35 dpi (Fig. 3C). No signals were observed in the sections incubated with pre-immune normal serum (Fig. 3B, C). Furthermore, western blot results showed that Ts-TPx1 is a significant component of *T. spiralis* ML ES product, which reacted with sera from mice immunized with rTs-TPx1 (Fig. 3D).

### Specific serum IgG and intestinal mucosal sIgA responses to rTs-TPx1 immunization

**Fig. 4:**
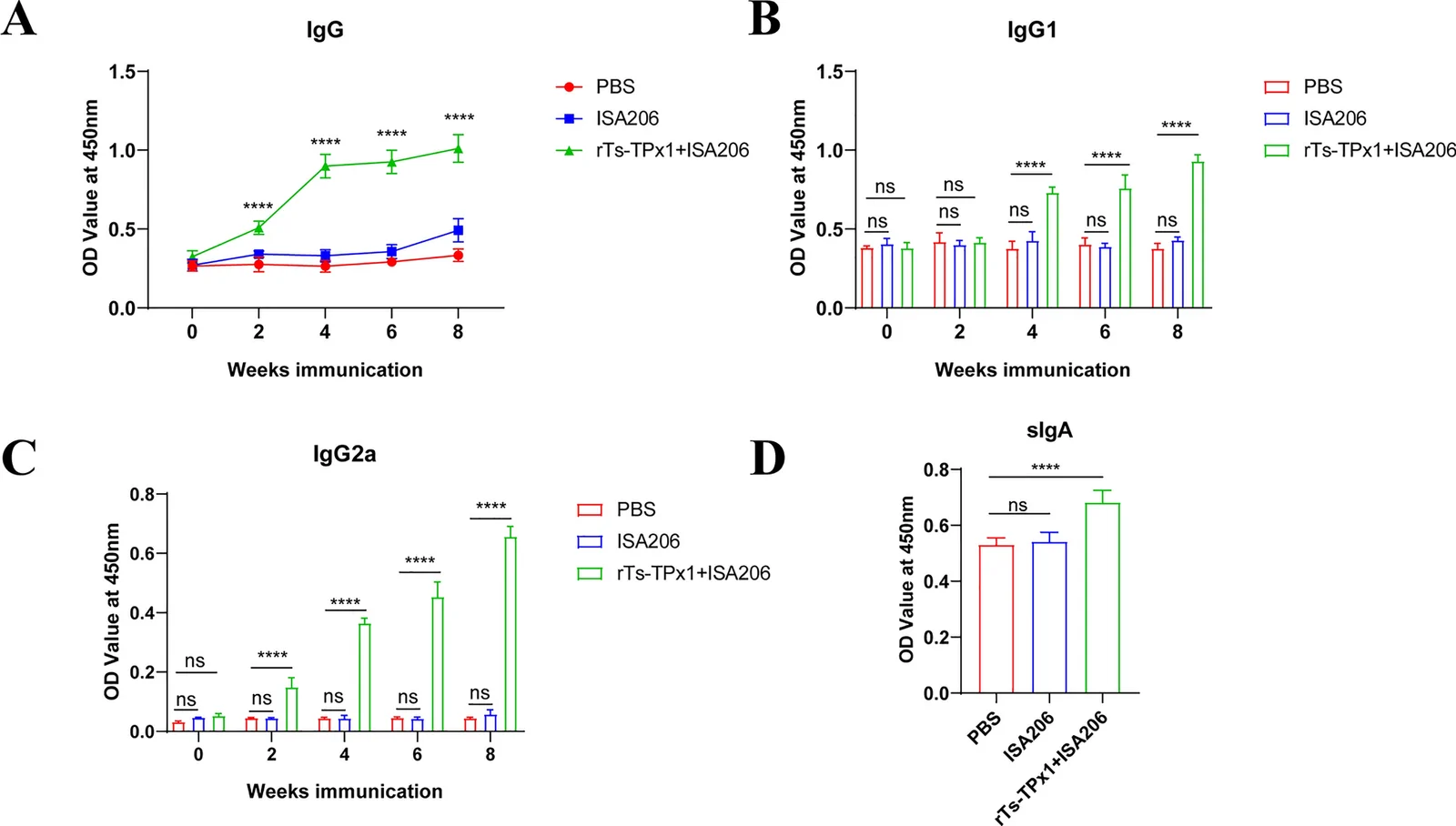
Specific antibody responses elicited by rTs-TPx1 immunization. The levels of anti-rTs-TPx1 IgG (**A**), IgG1 subclass (**B**), and IgG2a subclass (**C**) in the sera of mice were determined by ELISA using rTs-TPx1 (2 μg/ml) as coating antigen. Differences were analyzed by two-way ANOVA. **D** rTs-TPx1-specific sIgA levels in the enteral eluent of mice were measured by ELISA. Differences were analyzed by one-way ANOVA. The values shown are shown as the mean ± SD (*n* = 6 biological replicates). **** *P* < 0.0001, ns represents no significant difference, compared to the PBS group

To determine anti-Ts-TPx1 humoral antibody responses, the titers of rTs-TPx1-specific IgG and its subclasses (IgG1 and IgG2a) in the sera of vaccinated mice were quantified by rTs-TPx1-ELISA. The serum anti-rTs-TPx1 total IgG level in immunized mice was gradually increased upon vaccination with rTs-TPx1 (Fig. 4A), confirming that rTs-TPx1 was highly immunogenic and induce a humoral immune response in host. Anti-rTs-TPx1 IgG responses were not detected in mice vaccinated with the ISA 206 or PBS alone (Fig. 4A). Moreover, levels of  IgG1 and IgG2a in rTs-TPx + ISA206 group were significantly higher than both control groups. (Fig. 4B, C). Notably, the IgG1 levels at 4, 6 and 8 weeks after the second and third immunization were higher than IgG2a levels (Fig. 4B, C), indicating that immunization with rTs-TPx1 triggered a Th2-predominant Th1/Th2-mixed immune response.

To assess the enteral mucosal sIgA response to rTs-TPx1 immunization, rTs-TPx1-specific sIgA were determined using ELISA. The results showed that the specific sIgA levels in rTs-TPx + ISA206 group but not the ISA206 group were evidently higher than that in the PBS control group at 6 dpi (Fig. 4D), suggesting that rTs-TPx vaccination elicited an evidently specific intestinal mucosal sIgA response.

### Cytokines response elicited by rTs-TPx1 immunization

**Fig. 5:**
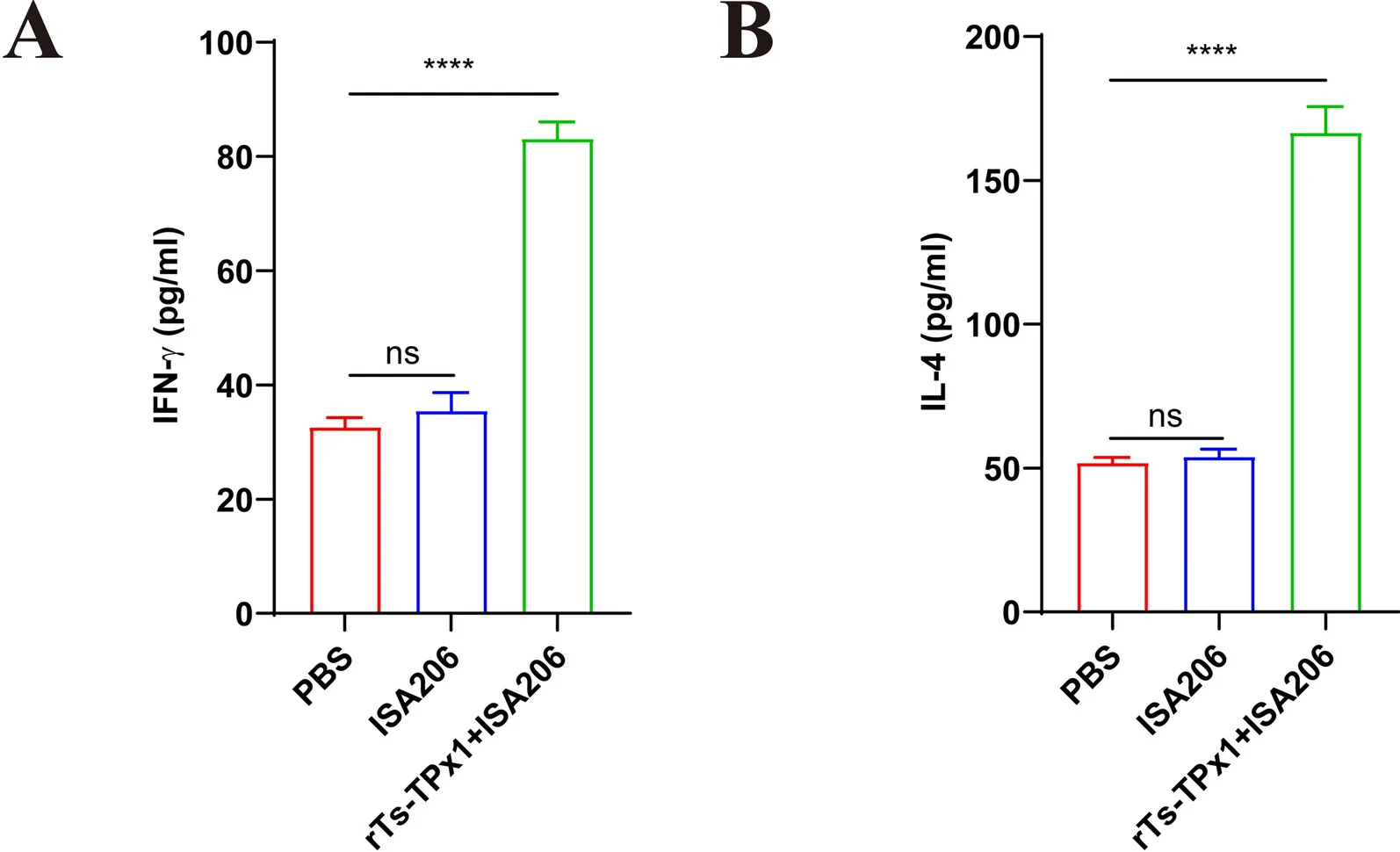
Analysis of cytokine secretion from splenocytes after rTs-TPx1 stimulation in vitro. The concentrations of IFN-γ (**A**) and IL-4 (**B**) were detected in the supernatant after the splenocytes were stimulated with 5 μg/ml rTs-TPx1 for 48 h. The values shown are shown as the mean ± SD (*n* = 5 biological replicates). Differences were analyzed by one-way ANOVA, **** *P* < 0.0001, ns represents no significant difference, compared to the PBS group

To ascertain that a mixed Th1/Th2 response was elicited, the levels of Th1/Th2 cytokines (IFN-γ and IL-4) in splenocyte culture supernatants were measured by ELISA. Compared to the PBS group, the levels of IFN-γ and IL-4 were significantly elevated in rTs-TPx1-stimulated splenocytes from mice in the rTs-TPx1 + ISA206 group. In contrast, rTs-TPx1 stimulation did not affect cytokine secretion by splenocytes from ISA206 alone-immunized mice (Fig. 5A, B). These findings further confirmed that a Th1/Th2-mixed response to rTs-TPx1 immunization was markedly evoked.

### Immune protection of rTs-TPx1 against *T. spiralis* challenge

**Fig. 6:**
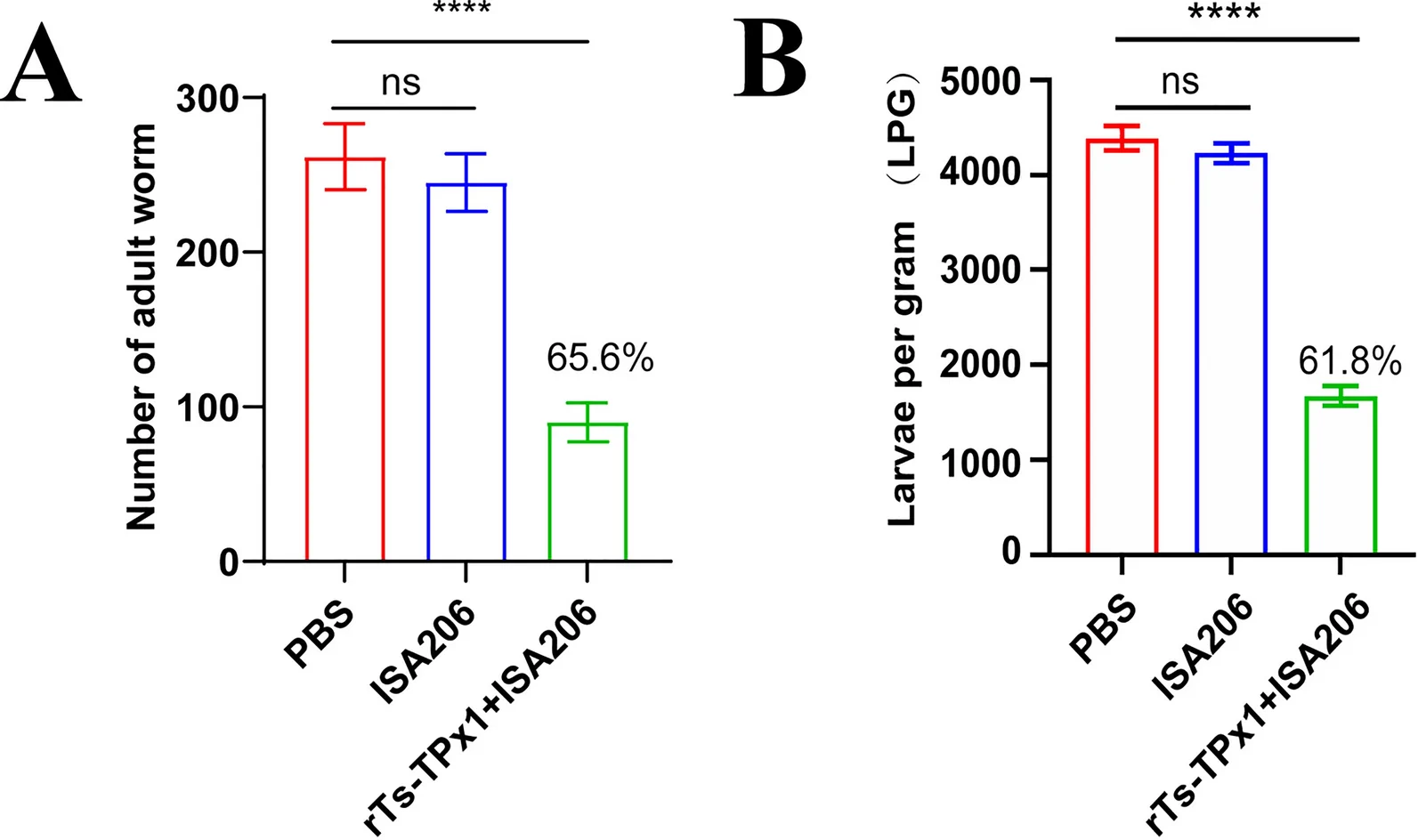
Immune protection induced by rTs-TPx1 immunization in mice after challenge with 300 ML of *T. spiralis*. **A** Number of adult worms recovered from intestines at 6 dpi. **B** Muscle larval burden (larvae per gram, LPG) in diaphragm muscles at 35 dpi. Results are shown as the mean ± SD (*n* = 6 biological replicates). Differences were analyzed by one-way ANOVA, **** *P* < 0.0001, ns represents no significant difference, compared to the PBS group

Six mice from each group were sacrificed at 6 days and 35 days after challenge to estimate the protective efficacy of Ts-TPx1 against *T. spirails* larval infection. Compared to mice in the PBS group, the rTs-TPx1 immunization group exhibited a 65.6% intestinal adult worm reduction at 6 dpi and a 61.8% reduction in ML burden at 35 dpi following larval challenge (Fig. 6A, B). However, no significant reduction in the ML and AD6 burdens was detected in mice immunized with the adjuvant ISA 206 alone in comparison with the PBS group. These results demonstrated that immunization of mice with rTs-TPx1 triggered significant immune protection against *T. spiralis* larvae challenge.

### Intestinal and muscle histopathological changes in infected mice

**Fig. 7:**
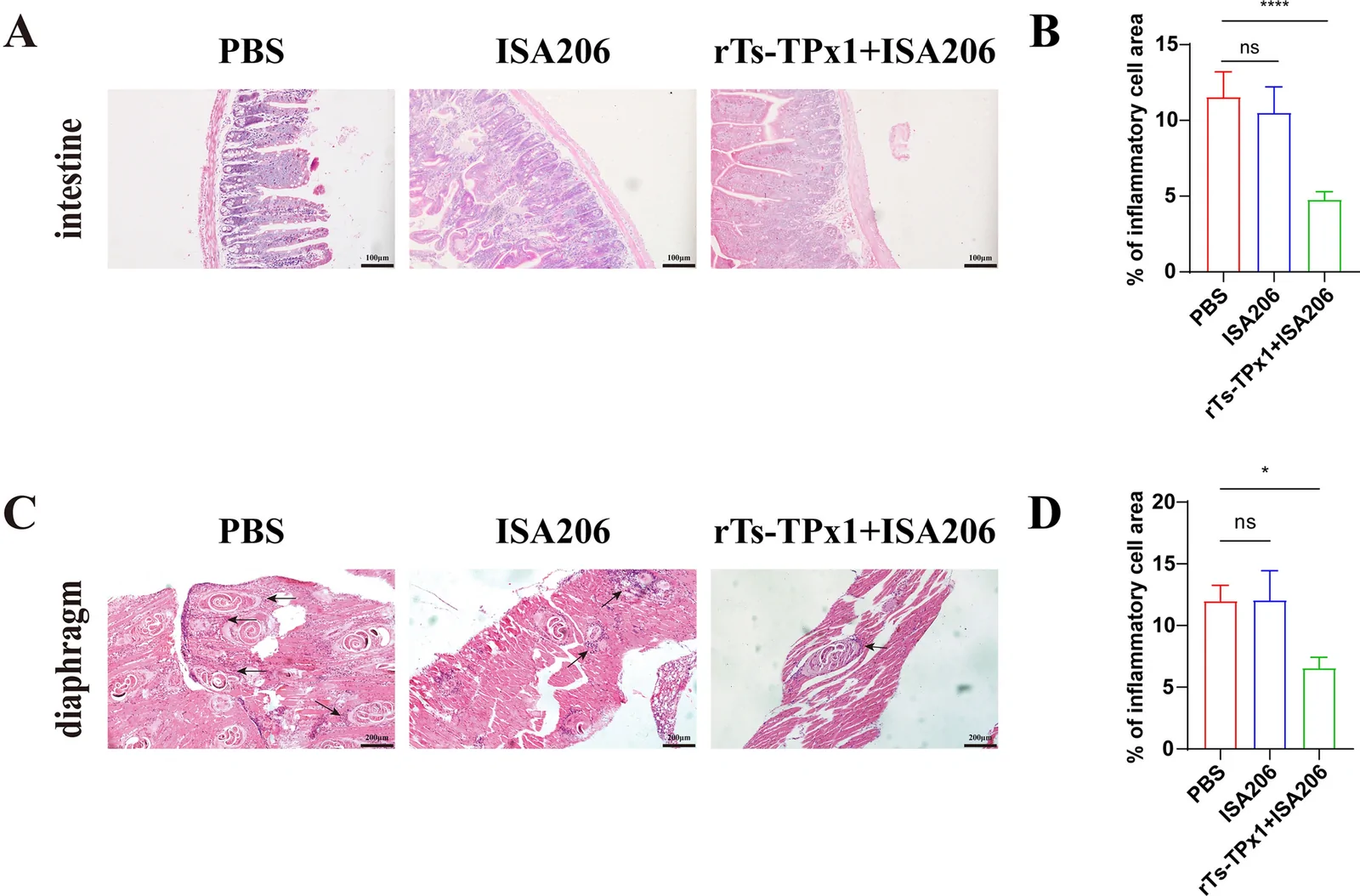
Histopathological changes in intestine and diaphragm muscles in infected mice. **A** Representative images of photomicrographs of fixed intestine sections after staining with HE. **B** Quantification of intestinal mucosal inflammatory cells in intestine sections. **C** Representative images of photomicrographs of fixed diaphragm muscle sections after staining with HE. Arrows indicate inflammatory cell infiltration surrounding the encapsulated larvae. **D** Quantification of inflammatory cells around encapsulated *T. spiralis* larvae on muscle sections. Results are shown as the mean ± SD (*n* = 5 biological replicates). Differences were analyzed by one-way ANOVA, * *P* < 0.05, **** *P* < 0.0001, ns represents no significant difference, compared to the PBS group. Intestine scale bars: 100 μm, muscle scale bars: 200 μm

Histopathological changes of the intestines and diaphragm muscles of mice were investigated at 6 and 35 dpi, respectively. HE stainning showed that an obvious destruction of villus structure and marked inflammatory cell infiltration in the intestinal tissue of mice challenged with larvae infection at 6 dpi (Fig. 7A). In contrast, the rTs-TPx1-immunized group exhibited improved villus structure and reduced intestinal inflammation, as evidenced by a remarkable lower number of infiltrating cells in the intestinal mucosa compared with the PBS group (Fig. 7A, B). Similarly, at 35 dpi, the number of inflammatory cells around encapsulated larvae in the diaphragm muscles of mice following rTs-TPx immunization was clearly reduced compared to mice in the PBS group (Fig. 7C, D). These results suggested that rTs-TPx immunization largely hindered larval invasion of the intestinal mucosa and alleviated muscle inflammation.

### rTs-TPx1 immunization increased the numbers of goblet cells and promoted Muc2 expression in infected mice

**Fig. 8:**
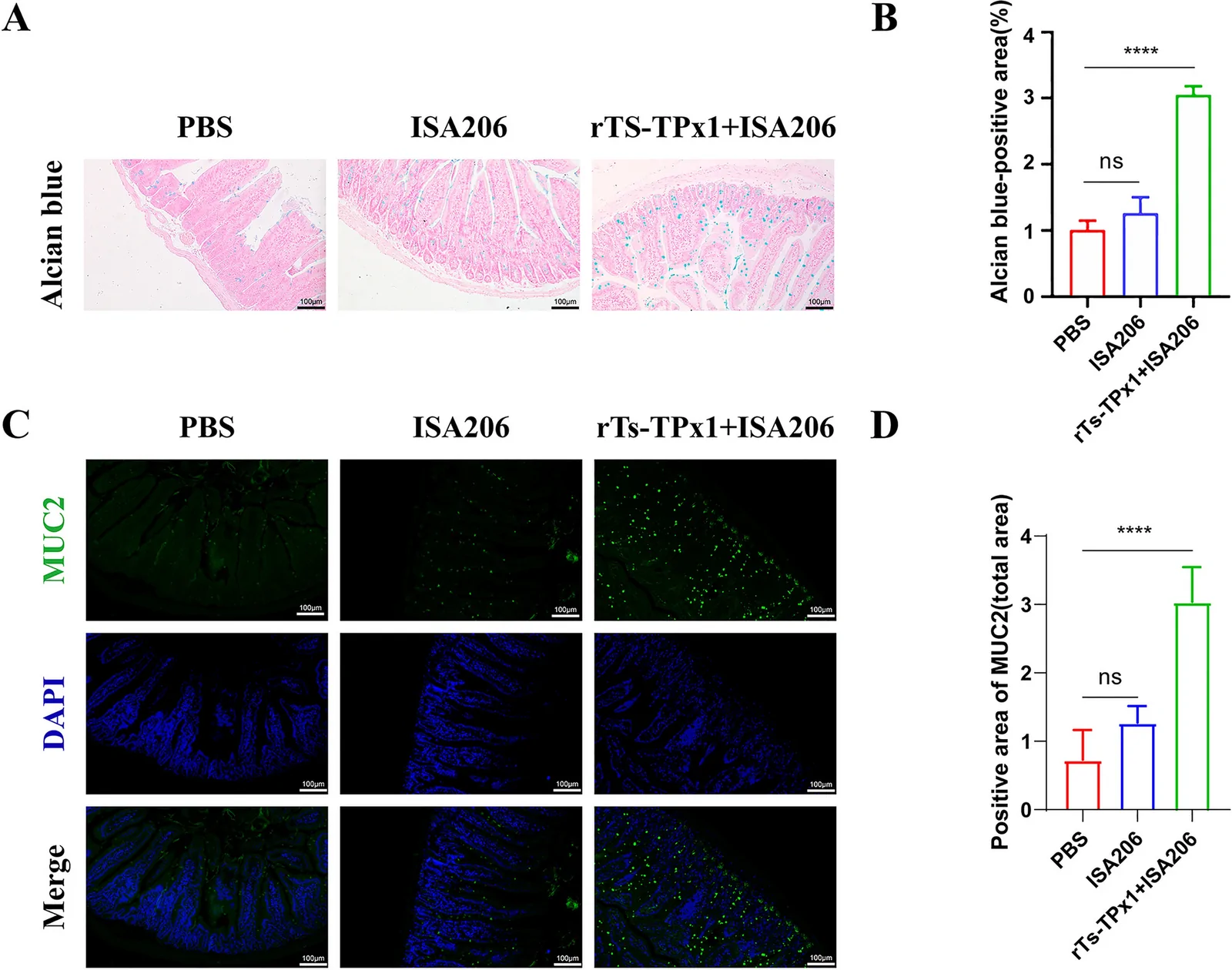
Effect of rTs-TPx1 immunization on intestinal goblet cells and Muc2 expression in mice. **A** Representative images of intestine Alcian blue staining. **C** Representative images of Muc2 protein immunofluorescence assay. Quantitative analysis of positive area of Alcian blue staining (**B**) and Muc2 protein (**D**). Results are shown as the mean ± SD (*n* = 5 biological replicates). Differences were analyzed by one-way ANOVA, **** *P* < 0.0001, ^ns^ represents no significant difference, compared to the PBS group

Mucins secreted by intestinal goblet cells contribute to defense against a number of enteric infections. In this study, Alcian blue staining was performed to simultaneously visualize goblet cells and mucin expression in intestinal sections. The results showed that the rTs-TPx1-immunized group exhibited a substantial increase in goblet cell density, along with prominent blue-stained acidic mucin accumulation in the intestinal mucosa (Fig. 8A, B). Moreover, IFA results indicated that mice immunized with rTs-TPx1 exhibited enhanced Muc2 expression in comparison with the mice administrated with PBS or ISA206 alone (Fig. 8C, D), which might contribute to improved mucosal barrier integrity and local immune protection against *T. spiralis* invasion.

## Discussion

At present, vaccines for controlling *T. spiralis* infection in humans and pigs are still in the stage of laboratory research [14]. In recent years, several antigens from different *T. spiralis* lifecycle stages have been expressed, and their immune protection potential has been investigated, such as proteases identified by cDNA library and immunoproteomics screening, as well as low-molecular weight proteins from the *T. spiralis* draft genome with high expression at the ML stage [10, 24]. However, these vaccine candidates have failed to achieve complete or even 80% protection against experimental infection in mice and pigs, with an average protection rate of approximately 50%. Thus, the identification of potential vaccine candidates against infection with *Trichinella* spp. remains a significant challenge. To address this, we evaluated the immunogenicity and protective efficacy of the excretory-secretory antigen Ts-TPx1. Our findings demonstrated that immunization with rTs-TPx1 elicited robust systemic and mucosal immune responses, conferring significant reductions in adult worm and muscle larval burdens, diminished infection-induced pathology, and enhanced intestinal barrier function. Collectively, these findings established Ts-TPx1 as a highly promising vaccine candidate for combating trichinellosis.

Thioredoxin peroxidase (TPx) is a validated immunogen against *Schistosoma mekongi* [16], and TPx2 from *T. spiralis* has been shown to induce T-helper 2 (Th2) immune response in mice by directly promoting alternatively activated macrophages [19]. The expulsion of worms is CD4^+^ T cell-dependent and requires Th2 cytokines [24]. We, therefore, hypothesized that Ts-TPx1 constitutes a viable vaccine target. Western blot and IFA analyses revealed constitutive expression of Ts-TPx1 in both muscle larvae (ML) and adult worms (AD6), consistent with an earlier report [18]. Notably, the Ts-TPx1 was localized predominantly to the tegument of both stages. This surface exposure equips the parasite to resist host-derived oxidants and simultaneously presents the enzyme to the immune system, satisfying the prerequisites for a protective antigen. Vaccination of mice with recombinant Ts-TPx1 (rTs-TPx1) and subsequent oral challenge reduced intestinal adult worms by 65.0% and muscle larvae by 61.8% relative to PBS controls. The protection rate conferred by rTs-TPX1 antigen is higher than that of characterized recombinant antigens of *T. spiralis*, such as protease and protease inhibitor [14], confirming Ts-TPx1 as a promising candidate for vaccination against infection.

To further assess the protective immunity elicited by rTs-TPx1 in mice, both humoral and cellular immune responses were evaluated. The results demonstrated that immunization with rTs-TPx1 induced a systemic mixed Th1/Th2 response, characterized by elevated levels of the Th1 cytokine IFN-γ and the Th2 cytokine IL-4, both of which contribute critically to protection against *T. spiralis* infection [26]. Th2-type cytokines such as IL-4 play a crucial role in inducing mast cell and goblet cell hyperplasia with mucus production, ultimately resulting in worm expulsion [27]. The Th1 component, characterized by cytokines like IFN-γ, is also important for activating cellular immune responses with enhance cytotoxic killing to exert its protective effects against parasite infection [24]. Thus, the observed Th1/Th2 mixed immunity induced by rTs-Tpx1 provides a multi-pronged attack against the complex parasite life cycle, directly contributing to the robust reduction in muscle larvae. IL-10 is a key regulator of protective immune responses against *T. spiralis* in vivo. In the absence of IL-10, mice were highly susceptible to a primary *T. spiralis* infection and significantly reduced numbers of mucosal mast cells [28]. Mice immunized with vaccine candidates of *T. spiralis* consistently upregulate IL‑10 which correlates with significant reductions in larval burden [24]. IL-17A secreted by Th17 cells play an important role in protecting against mucosal infection [29]. IL-17 may contribute to jejunal muscle contractility in mice with *T. spiralis* [30]. Future studies should focus on characterization of the T-helper cell subsets (Th1, Th2, Th17, Treg) and their associated cytokine profiles to further delineate effector mechanisms induced by rTs-TPx1. Since T cell cytokines regulate B cell antibody class-switching, the resulting immunoglobulin isotypes can serve as markers of Th1/Th2 responses.. In the mouse strain used (e.g., ICR and BALB/c), a Th1‑type response is indicated by elevated IgG2a, whereas a Th2‑type response is reflected by elevated IgG1 [31]. In this study, rTs-TPx1 triggered a mixed IgG1/IgG2a antibody profile, with IgG1 being predominant. The high titers of specific IgG1 antibodies indicated a robust Th2-driven adaptive immune response. IgG1 plays a key anti-parasitic role by binding to functional sites or critical surfaces of Ts-TPx1, thereby directly inhibiting its enzymatic activity and leaving the parasite susceptible to host oxidative attack. Moreover, IgG1 exhibits high affinity for Fcγ receptors on macrophages, neutrophils, eosinophils, and NK cells, and is instrumental in antibody-dependent cell-mediated cytotoxicity (ADCC), a mechanism effective against large multicellular parasites [32, 33]. Previous reports have shown that neutrophils, in combination with monospecific antibodies, can inhibit larval molting in *Onchocerca volvulus* [34], and anti-*Trichinella* antibodies have been found to impede parasite growth in ADCC assays with monocytes [35]. Falduto *et al*. demonstrated that particularly eosinophils and neutrophils, are highly effective at killing newborn larvae through ADCC [36]. In this study, IFA analyses indicated that Ts-TPx1 is localized on the cuticle of both muscle larvae and adult worms. Based on this observation and its function, we hypothesize that the protein is also present on the surface of newborn larvae (NBL). If confirmed, this surface localization would allow Ts-TPx1‑specific IgG1 antibodies binds to the surface of newborn larvae to destroy migrating NBL via ADCC. However, further investigations, such as immunolocalization on live NBL and ADCC assay, are required to directly demonstrate the presence of Ts-TPx1 on the NBL surface and to validate its functional involvement in ADCC‑mediated protection. Intestinal nematodes invade the host through the intestinal mucosa, making mucosal immunity the first line of defense against parasitic entry. In this study, we further evaluated intestinal sIgA responses and goblet cell activity. sIgA plays a critical role in mucosal defense by neutralizing pathogens and ADCC, thereby preventing parasitic establishment at mucosal surfaces [37]. Previous studies have demonstrated that parasite-specific sIgA can inhibit *T. spiralis* larval colonization and promote expulsion in mice [38]. Thus, the observed increase in Ts-TPx1-specific sIgA suggested its involvement in protective mucosal immunity against *T. spiralis* infection. *T. spiralis* infection typically induces a strong IgE response, which cooperates with mast cells to promote intestinal infective larvae and adult worm expulsion from the intestines of vaccinated animals. IgE activating mast cells and eosinophils have the potential to directly kill larvae [39, 40]. One limitation of this study is that we did not directly measure Ts-TPx1‑specific IgE or assess mast cell activation. The absence of these data prevents us from fully delineating the immunoprotection mechanism. Nevertheless, the robust Th2 polarization (IL-4 and IgG1) and the reduction in worm burden we observed are consistent with an IgE‑mediated pathway. Future studies will investigate roles of rTs-TPx1 elicited specific IgE in protective immunity against *T. spiralis* infection.

Furthermore, immunization with rTs-TPx1 significantly increased intestinal goblet cell numbers and Muc2 expression while reducing intestinal inflammation. Goblet cell-derived mucins constitute an essential innate barrier against enteric helminths by expelling the parasite and limiting worm movement. Deficiency of Muc2 leads to spontaneous inflammation and susceptibility to infection [41]. Up-regulation of Muc2 is commonly associated with resistance to parasites, such as *T. spiralis*, *Nippostrongylus brasiliensis*, and *Trichuris muris* [42]. Specifically, mice with a robust rTs‑TPx1‑specific immune response showed significantly higher Muc2 expression and less mucosal disruption in the small intestine compared to non‑immune controls. Furthermore, the reduction in larval burden following rTs‑TPx1 vaccination correlated positively with Muc2 expression. These findings indicate that an rTs‑TPx1‑specific immune response not only limits parasite establishment but also actively preserves the mucus layer, thereby maintaining gut barrier function during *T. spiralis* infection. Further experiments are warranted to explore whether TPx1-induced immune signaling modulates goblet cell biology and mucin production, potentially linking pathogen recognition to mucosal barrier alterations. These findings provide new evidence that rTs-TPx1 vaccination enhances innate intestinal defense mechanisms. Notably, inflammatory cell infiltration in both intestinal and muscle tissues was markedly reduced at 6 and 35 dpi in rTs-TPx1-immunized mice compared with adjuvant or PBS controls. This results indicated that vaccination not only strengthened mucosal barrier functions but also effectively mitigated infection-induced tissue inflammation.

*T. spiralis* exists a complex life cycle involving different developmental stages (adult worms, newborn larvae, and muscle larvae). In this study, the immunization of mice with rTs-TPx1 did not confer complete immune protection against *T. spiralis* challenge infection. Recent studies have indicated that combining antigens from different life cycle stages may induce immunity against the entire parasitic cycle to reduce intestinal worm establishment, kill migrating newborns, and prevent larval encapsulation [43, 44]. Therefore, the development of multi-antigen or “cocktail” vaccines containing multiple protective antigenic epitopes from diverse *T. spiralis* developmental stages should be evaluated for their immunoprotective effects in future experimentes, with the goal of  simultaneously elicting antibody and cellular responses.

## Conclusions

This study demonstrated that immunization with rTs-TPx1 conferred partial protection against *T. spiralis* challenge in mice. The vaccine elicited a systemic Th2-biased Th1/Th2 mixed immune response and enhanced local intestinal mucosal immunity. Active immunization resulted in significant protection against *T. spiralis* challenge. These findings demonstrated that rTs-TPx1 induced a multifaceted protective immune response and presented a strong, antigen-specific vaccine candidate for controlling *Trichinella spiralis* infection.

The protective efficacy of a vaccine is influenced by factors, such as immunization dose, number of administrations, adjuvant and delivery route. Further optimization, including evaluation of alternative routes (e.g., oral or intranasal) and adjuvant, will be necessary to enhance protection. Currently, vaccine experiments with partial protection in *T. spiralis* are generally based on acute challenge models, and durable memory T and B cell responses are essential for vaccine-induced immunity, providing long-lasting protection against infectious diseases. Future vaccine development should be systematic evaluation of immune memory using extended challenge intervals and memory B and T cell populations. In addition, elucidating the precise immunological mechanisms underlying Ts-TPx1-induced protection will require integrated investigation across vaccinology and immunobiology. A deeper understanding of these mechanisms will support the rational design of more effective and broadly protective anti-helminth vaccines.

## Data Availability

Data supporting the main conclusions of this study are included in the manuscript.

## Abbreviations

ML: Muscle larvae
Ads: Adult worms
TPx: Thioredoxin peroxidases
Ts-TPx: *T. spiralis* Thioredoxin peroxidases
rTs-TPx: Recombinant
dpi: Post infection
cds: Coding sequence
SDS–PAGE: Sodium dodecyl sulfate–polyacrylamide gel electrophoresis
PVDF: Polyvinylidene difluoride
IFA: Immunofluorescence assay
LPG: Larvae per gram
HE: Hematoxylin and eosin
Muc2: Mucin 2
OD: Optical density
aa: Amino acids
